# Phosphate of Lime—Suggestions as to Its Use by Dentists

**Published:** 1872-10

**Authors:** J. E. Cravens


### Phosphate of Lime—Suggestions as to its Use by Dentists.

BY J. E. CRAVENS.

Philosophizing in a general way upon a subject tends to leave readers, in reference to it, very much in condition of the mariner without a compas.

The usually purchasable article of phosphate of lime as we obtain it from the druggist is the powdered preparation. The attention of the dental profession has for some time been directed toward experiments, looking to the arresting of caries of the teeth through medical agencies. If from systematic cause the consistency of the dental organs is reduced from a healthy density to an impoverished, want of ability to resist such decomposing agents as attack them; and if we are aware of the nature of that depravity, and the destructive changes which produce it, then there should be some means of repairing the damage throughout the same channel. Figuratively, the body is but a railroad, and all trains upon it do not run in the same direction. Where a will exists there is pretty sure to be a way to the accomplishment. It has been, long enough, definitely known that the deficient element in teeth highly susceptible to action of dental caries, is the earthy matter or lime salts; and, that when the proper proportion of this principle exists, we find strong, healthy teeth, fully able to resist decay if but assisted by reasonable cleanliness. To correct this depravity then it is necessary to replenish with material similar to that which has been lost. It is true enough that the various grains, such as wheat, oats, etc., contain sufficient nourishment of this particular character to supply under peculiarly favorable circumstances, all the needs of the body. At any rate such seems to have been the original design in the creation. But man was granted the perogative of choosing from all the things upon the earth and in it. He started out by making a bad choice, and being the tural possessor of imagination, ingenuity, and numberless appetites, he chose to cater especially to his senses of taste, sight and smell; consequently we peel our apples and potatoes, and condemn corn-bread as a vulgarity, bolt our floor and turn up our proboses at oat-meal as fit only for horses.

The Scotch common people subsist entirely upon oat-meal for a bread stuff. And if any dentist is spoiling for a *hard pull* I commend him to the "land o' cakes." Oat meal contains more phosphate of lime than any other substance from which bread is made, and to this diet is ascribed the characteristic hardness of Scotchmen's *heads*, and bones generally. A highlander's teeth will resist decay, if somebody else will clean them for him. If a patient is to be treated for depravity of phosphate of lime in the teeth, the desired result might be accomplished in *time* by a rigid course of dieting, provided the patient should live long enough (!) and even then the dentist would be apt to find life growing monotonous ere he reached a satisfactory result. If a man's digestive aparatus is vigorous and healthy, and his absorbents having never been imposed upon, stand in open mouthed credulity, ready to receive anything that the stomach puts its stamp upon, that man's teeth might be benefited some, by confining himself to cracked wheat, unbolted floor, etc. But such individuals have very little use for the services of the dentist, and rarely indeed, if ever, for treatment for the affection in question. Those persons who do need our services in this direction are those either suffering from indigestion and a want of assimilation—in short, dyspeptics—or females in state of pregnancy, or lactation, and individuals whose bones are illy nourished from any other cause whatever. I believe that in ninety per cent. of cases of rachitis, the teeth, if examined, will be found sadly deficient in lime salts. Although debilitated patients may receive some benefit from phosphate of lime in regular alimentation, yet the process would be so slow as to give in most cases no tangible evidences of good; and the patient becoming tired of a monotonous bill of fare, would break through your guards, and as before remarked, fall to satisfying his senses of sight, smell and taste. Verily, the appetite for the flesh-pots is strong. These facts have not been overlooked heretofore by the investigating men of our profession, on the contrary they stuck a pin there, and sought to reach the goal by a shorter route, and certainly a correct one, i. e. medication. Pulverulent phosphate of lime was administered to patients, and gave the most flattering results, in all cases where the patients could take it for a proper length of time; but a trouble arose here that in many cases proved an insurmountable barrier, and has remained so until quite recently. The obstacle heretofore existing as before the success of phosphate of lime, was that in probably nine tenths of cases the patients after a few weeks at the outside conceived a violent dislike for their medicine, amounting to actual disgust at the sight of the powder. Why was it so? I have already said this was the short and correct road: *but it needed corduroying*, and there is where the failure was. However, this is the age of progress; and whether they originated the idea or not, still to the French M. Ds. belong the honor of applying the corduroy; viz: they digested the phosphate of lime in lactic acid, and administered it in a syrup. This syrup calcis lacto phosphatum; has a taste and smell very similiar to the juice of the common goosberry. It is given in doses of from a teaspoon to a tablespoon full, once to thrice *per diem*, generally after meals as it greatly facilitates digestion. The patient can easily receive from ten to thirty grains daily, as may be desirable. The peculiar virtue of this preparation is that it can be taken in nearly all cases with perfect regularity of dose, and cause no revolting emotions on the part of the patient. The appetite is increased because digestion is promoted; and what benefit is derivable from proper dieting, the patient is better able to receive; because assimulation has restored to it a healthy tone. In short the lacto phosphate of lime is a tonic of rare virtues, it goes straight to the mark, does its work well, is prompt in effect, and is withal quite agreeable to the taste. One objection to this preparation is when the patient is impecunious. It is anything but cheap, owing to the extravagant price of lactic acid. The laboratory process is of a delicate, complicated nature, consequently only manufacturing chemists prepare it. Now, perhaps a few items in reference to how experimentors were led to adopt this preparation of lime salt would be of interest.

The acidity of the gastric juice is due to the presence of lactic acid. The proper solvent of phosphate of lime is lactic acid. Lactic acid will only take up or digest, I think, from two to five per cent. of the pulverulent salt, and this in the test tube, with the acid officinal and the temperature correct. The gastro-lactic acid is much diluted by being only one of the several constituents of the stomach fluid, being in proportion of only about two parts in a thousand, so that when the powdered phosphate of lime is introduced into the stomach, it lies there next to insoluble, or is carried forward by peristaltus and ejected at the terminus of the alimentary canal; there being, as already stated, exceptional cases of extraordinary digestive power, in which the patient received corresponding benefit. When the stomach refuses to digest the powder then is experienced the revolt against the medicine, and the doctor must stop. From these facts was conceived the idea of predigesting the phosphate in lactic acid in the laboratory, which was done, and a syrup prepared which was to be a vehicle for introducing the lacto phosphate of lime into the stomach. This preparation is now extensively used in France by the medical profession, and very recently indeed have American physicians recognized its merits, and many now administer it. It is a remedial agent that especially suits the dentist's wants, and I trust that the profession throughout this country will be foremost in adopting it, as they have done in regard to every good thing which has been heretofore brought to their notice. Ask your druggist to procure for you the syrup lacto, phosphate of lime.

